# OLDER VETERAN EXPERIENCES OF TECHNOLOGY USE DURING A MULTICOMPONENT TELEREHABILITATION PROGRAM

**DOI:** 10.1093/geroni/igae098.2563

**Published:** 2024-12-31

**Authors:** Michelle Rauzi, Melissa Tran, Cory Christiansen, Meredith Mealer, Kathryn Nearing, Jennifer Stevens-Lapsley

**Affiliations:** Department of Veterans Affairs, Aurora, Colorado, United States; University of Colorado Anschutz Medical Campus, Aurora, Colorado, United States; University of Colorado Anschutz Medical Campus, Aurora, Colorado, United States; University of Colorado Anschutz Medical Campus, Aurora, Colorado, United States; University of Colorado Anschutz Medical Campus, Denver, Colorado, United States; University of Colorado Anschutz Medical Campus, Aurora, Colorado, United States

## Abstract

Less than half of U.S. military Veterans meet physical activity recommendations despite the well-established health benefits of regular physical activity. While changing physical activity behaviors is challenging, prior studies demonstrate that it is possible. Older adults are using technology to aid in such behavior change, however, research that explores the mechanisms of how technology can aid in behavior change is lacking. Thus, the purpose of this secondary, convergent mixed methods study was to explore how older Veterans engaged with technologies that were used during a multicomponent telerehabilitation program. The study included Veterans aged ≥60 years with ≥3 chronic medical conditions and physical function limitation. Quantitative data were collected during the primary randomized controlled trial, and qualitative data were collected via individual interviews following the telerehabilitation program. Data were merged and then categorized by level of technology engagement. Key similarities and differences between the high and low technology engagement groups were identified in five domains: satisfaction with the virtual environment, coping self-efficacy, perceptions of Annie (automated text messaging platform), experiences using the activity monitor, and self-management skills. Results revealed three key findings that help explain why older Veterans chose whether to engage with the technologies used during our behavior change program. These findings relate to technology self-efficacy, integration of the technologies into intervention, and technology application to self-management skills. Study results can help with the integration of similar technologies into physical rehabilitation programs. Further study is warranted to understand additional factors and mechanisms that influence technology engagement in healthcare.

